# Anthropomorphic Design: Emotional Perception for Deformable Object

**DOI:** 10.3389/fpsyg.2018.01829

**Published:** 2018-10-02

**Authors:** Jung Min Lee, Jongsoo Baek, Da Young Ju

**Affiliations:** ^1^Technology and Design Research Center, Yonsei Institute of Convergence Technology, Yonsei University, Incheon, South Korea; ^2^Yonsei Institute of Convergence Technology, Yonsei University, Incheon, South Korea

**Keywords:** anthropomorphism, emotional interaction, deformable object, human–computer interaction, user experience

## Abstract

Despite the increasing number of studies on user experience (UX) and user interfaces (UI), few studies have examined emotional interaction between humans and deformable objects. In the current study, we investigated how the anthropomorphic design of a flexible display interacts with emotion. For 101 unique 3D images in which an object was bent at different axes, 281 participants were asked to report how strongly the object evoked five elemental emotions (e.g., happiness, disgust, anger, fear, and sadness) in an online survey. People rated the object’s shape using three emotional categories: happiness, disgust–anger, and sadness–fear. It was also found that a combination of axis of bending (horizontal or diagonal axis) and convexity (bending convexly or concavely) predicted emotional valence, underpinning the anthropomorphic design of flexible displays. Our findings provide empirical evidence that axis of bending and convexity can be an important antecedent of emotional interaction with flexible objects, triggering at least three types of emotion in users.

## Introduction

Shapes are closely related to emotion. Takahashi (1995) researched pictorial perception, assessing person–object relations. According to the study, aesthetic characteristics, such as lines and textures, are related to the perceptual experience, interacting with expressive emotions, such as anger, happiness, serenity, disgust, sadness, and femininity. A study on shape has also been conducted, finding that shape evokes emotion in people. A certain shape can be linked with the adjectives that describe it; thus, a circular shape is related to the adjectives sad, clumsy, and passive while a triangular shape evokes a sharp and dangerous feeling. On the other hand, a quadrilateral shape induces a heavy and strong feeling in participants. Here, with the underlying precondition that shape plays a role in person–object relations, this paper assesses whether a shape anthropomorphizing human posture interacts significantly with emotion.

In recent decades, there has been an increasing number of studies on user experience (UX) and user interface (UI) for deformable displays. With “anthropomorphism” as the philosophy of design, it is necessary to implement emotional interaction between humans and deformable displays to provide positive implementation of UX. Anthropomorphism, assigning human characteristics such as emotion to a non-human object, enables users to be familiar with the deformable display since people unconsciously derive emotional stability from things that are similar to themselves. Herein, this paper examines which functions would be appropriate to implement on these personified flexible devices in a theoretical framework, particularly focusing on interaction between emotional input and output.

## Literature Review

### Anthropomorphism

According to research, people unconsciously tend to be attracted to things that are similar to themselves (Berger and Bradac, 1982). In the uncertainty reduction theory, familiarity plays a crucial role in relationship development both among humans and between humans and devices (Berger and Bradac, 1982). Indeed, Epley et al. (2007) argued that humanlike entities implement more familiar, explainable, or predictable qualities than do non-humanlike entities. According to Reeves and Nass’ (1996) media equation theory, people tend to equate media (x) with real people (y) as if they were virtually experiencing real people or places. Thus, it is important to “give computers some personality” for the successful design of interactive technical products (Reeves and Nass, 1996).

**FIGURE 1:**
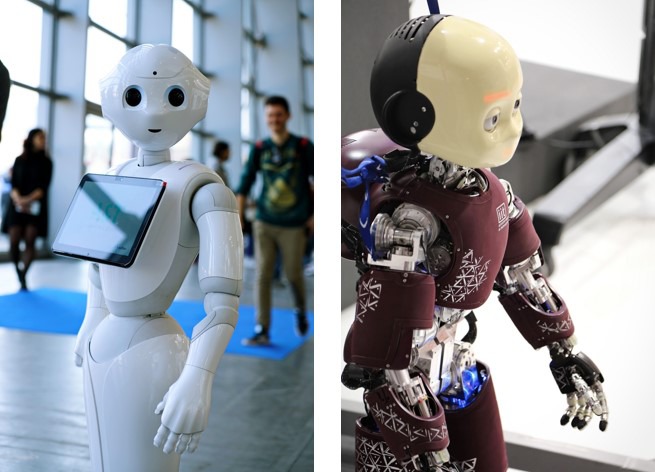
Anthropomorphism in robotics. Pepper (Left) and iCub (Right). Images, used under license from MikeDotta/Shutterstock.com.

Currently, anthropomorphism is extensively researched, particularly in the field of humanoid robots or human–robot interaction (HRI). Indeed, in the field of HRI, it has widely been found that anthropomorphized technologies, in the form of both humans and animal creatures, increase social interaction and support emotional bonding with humans (Li and Chignell, 2011; Yohanan and MacLean, 2012). For example, Softbank Robotics developed an emotionally interactive humanoid robot, Pepper, which identifies principal human emotions, changing mood, or behavior to interact with the users (Softbank Robotics, 2017). At the same time, iCub is another kind of humanoid robot that was developed at Istituto Italiano di Tecnologia. The iCub has its own sense of proprioception and movement as well as visual recognition capability developed via deep learning (The RobotCub Consortium, 2017) (see **Figure 1**).

Research on anthropomorphism in terms of interaction has not been limited to a humanlike appearance (Zlotowski et al., 2015). For instance, according to Kahn et al. (2006), six benchmarks elementary to humanlike interaction are autonomy, imitation, intrinsic moral value, moral accountability, privacy, and reciprocity. Turkle (2010) viewed behavior itself as the most crucial factor for anthropomorphic interaction with humans, and Wiese et al. (2017) recognized humanlike behavior as a critical factor underpinning social cognition in the human brain. Throughout this study, implementation of humanlike movement or behavior will be researched through usage of flexible displays.

At the same time, recent studies have attempted to evaluate a novel expression method applying principles of animation to technology. For example, Bates (1994) applied the animation theory “The Illusion of Life” (Thomas et al., 1995) developed by Walt Disney Animation Studios into the interactive agent. Likewise, van Breemen (2004) applied animation theory for the development of Lino and iCat, the UI robot. According to van Breemen (2004), development of emotionally interactive technologies is facing similar problems to those in the early stage of animation, contending that the principles of animation are applicable to interaction in robotics. For example, he viewed that easily recognizable poses enable users to easily identify actions. Other researchers have also employed or contrasted principles of classic animation, lifelike behavior of a character, with machine-like behavior of robots (Takayama et al., 2011; Ribeiro and Paiva, 2012; Saldien et al., 2014; Castro-González et al., 2016). However, this study focused on psychological means of bending a flexible display in terms of emotional interaction, eliminating other variables outlined by animation theory. This is because this study is exploratory research aiming to acquire new insights by finding phenomena common to all participants. Due to the characteristics of exploratory research, finding significant phenomena without explicit expectations (Schutt, 2011), there would be too many variables in animation theory to be covered in a single study. Therefore, it has been decided to limit the scope of the study to answer the question, “What emotion does the shape of an object provide to the user?” The result of the study itself can work as a framework for the researchers, designers, or manufacturers who explore the emotional interaction of flexible displays. Although the study focuses on a flexible display due to its familiarity, the results are applicable to the other kinds of objects with (1) rectangular shape, (2) distinguishable front and backside, and (3) technically deformable features.

### Flexible Devices

Flexible display, first conceptualized and prototyped by Xerox PARC (Palo Alto Research Company) in 1974, refers to a dynamic display that can be forced out of shape. Display-related companies, such as Nokia (Nokia Morph concept, 2008), Sony (OTFT-Driven OLED display, 2010), Samsung (Youm, 2013), and LG (OLED flexible display, 2013), have developed flexible displays (Noda et al., 2011; Mathur et al., 2013; Mone, 2013; Shao et al., 2015). Leading display manufactures registered patents on flexible displays, such as foldable and bendable displays, for portable devices (Rothkopf et al., 2014; Bae et al., 2018). These products are expected to permeate our lives, but, before we introduce these technologies, it is necessary to find the emotional value of such products.

There are two types of displays called flexible displays. The first is rigid and fixed in shape. Examples of this flexible display include the LG curved phone and Samsung Edge, which have glass material that works as a lower board and protects the display. The second type of flexible display is a dynamic display, which has an innovative form factor such that it can flexibly change its shape. For example, Samsung officially launched their flexible OLED display, called the Youm display, which was demonstrated at CES 2013 (Samsung, 2013). According to previous studies, these technologically available flexible displays have potential to trigger emotional interaction, enhancing usability (Lee et al., 2015; Strohmeier et al., 2016). Using anthropomorphism as a philosophy of design, this paper investigates the variables that convey emotional value to users by taking flexible display as a research domain.

#### UX Trend for Flexible Devices

Beyond stiff and brittle displays, there has been increasing research aiming to optimize UX and UI for flexible displays. Rendl et al. (2014) presented FlexSense, a thin, transparent, deformable surface. Through FlexSense, they proposed that a surface with a deformable display comprised a performative UI, providing a high degree of freedom in input control and applicable in various scenarios such as Photoshop, online maps, games, and education. Ahmaniemi et al. (2014) also proposed different methods of bending on a deformable display: zooming, image editing, reading, map information, navigation, browsing large amounts of information, quick reactions, and games. They argued that a dynamic control could be implemented on a flexible display that even requires high resolution.

Interaction methods of flexible displays have also been widely researched in recent years. Lee et al. (2010) examined the interaction method of flexible displays, such as bending, folding, and stretching, derived from plastic, paper, and elastic cloth. Likewise, Gomes et al. (2013) and Hemmert et al. (2013) researched possible interaction methods that could be applicable to future flexible devices. Warren et al. (2013), on the other hand, researched the bending interaction itself. They collected 36 bending gestures and investigated the preferred location and direction in which participants interacted. The UI of devices was also expected to be altered if the display became flexible in the future. Through FoldMe, Khalilbeigi et al. (2012) studied possible design spaces for a double-sided foldable display. Apart from finding possible ways to fold the displays, the authors designated possible UIs for the double-sided foldable display, indicating that it would be efficient for foldable multitasking, tool palettes, layers, and spin control.

#### Emotional Interaction of Flexible Devices

According to Triberti et al. (2017), technology designers should consider the emotional factors that align with users’ everyday lives, and these emotionally interactive technological products enhance loyalty and satisfaction and may promote happiness and well-being. Kwon and Ju (2018) also indicated that emotional experience of the customers, such as familiarity and comfort, should be considered for the customer centric design. Dawson et al. (2013) have studied the emotional interaction of deformable displays, finding that the flexible display provides simple but powerful gestural implications such as breathing, curling, crawling, ears, and vibration. Likewise, their study examined the emotional interaction of flexible displays but focused on the influence of complexity, direction (alignment), and convexity on emotional interaction for users. Through Bendi, a device that changes its upper and/or lower section, Park et al. (2015) indicated that the shape-changing device could be actively used to share emotions among users, facilitating both visual and tactile interaction. Their study also found that flexible devices enhance emotional communication between users. Moreover, Bailenson et al. (2007) investigated the emotional interaction between humans and tactile devices, although their study concentrated on tactile interaction with a virtual hand instead of flexible display interaction. Strohmeier et al. (2016) and Lee et al. (2015) also supported the existence of the emotional interaction of flexible displays through the Circumplex Model of Emotion developed by Russell (1980). The pattern, which implicates the sharing of emotion, was observed in each quadrant of the model.

Indeed, according to Lee et al. (2017), people tend to perceive flexible devices as humanlike, seeing the top as the head, the middle as the waist, and the lower part as the knees. When corner-bending was implemented, there was a tendency for participants to see the bending of the top corners as human arms and the bending of the bottom corners as legs. This result aligned with emotional studies that recognized the role of body language in expressing emotions (Atkinson et al., 2004; Clarke et al., 2005). Since previous studies were qualitative and particularly concentrated on the existence of emotional interaction instead of on humanlike bending, this study aims to find the standard tendency of participants regarding how they project humanlike bending on flexible displays.

#### Emotion Model and Flexible Devices

Extensive research has been conducted on the classification of emotion using facial expression and body shape (Darwin, 1872/1965; James, 1890; Ekman, 1965; Frijda, 1988; Jellema and Perrett, 2003; Atkinson et al., 2004; Clarke et al., 2005; Chouchourelou et al., 2006). Among various emotional models, Paul Ekman’s six basic emotions have been used extensively in research studies. According to Ekman (1999), there are six elementary emotions in terms of facial expression: happiness, sadness, anger, fear, disgust, and surprise. However, there are controversies regarding defining the elementary emotions into six groups with reports that it is difficult to either recognize particular emotions or replicate the study’s results. Indeed, Baron-Cohen et al. (1993) reported that the emotion “surprise” was not found in their study, and Oatley and Johnson-Laird (1987) found that surprise is amendable to cultural influences. Meanwhile, studies investigating expressions of emotions based on Ekman’s six basic emotions revealed confusion in discriminating surprise from other emotions. Particularly, a number of studies found surprise to be confused with fear (Calvo and Lundqvist, 2008; Tottenham et al., 2009; Recio et al., 2013; Jack et al., 2014). For instance, Jack et al. (2014), who researched dynamic expression of emotions, revealed that surprise is close to the emotion of fear. According to them, rather than being a “basic emotion,” surprise is a response, a reaction to something that has been unexpected. Herein, among Paul Ekman’s six basic emotions, only five were used to conduct the survey with surprise withdrawn from the list.

## Materials and Methods

**FIGURE 2:**
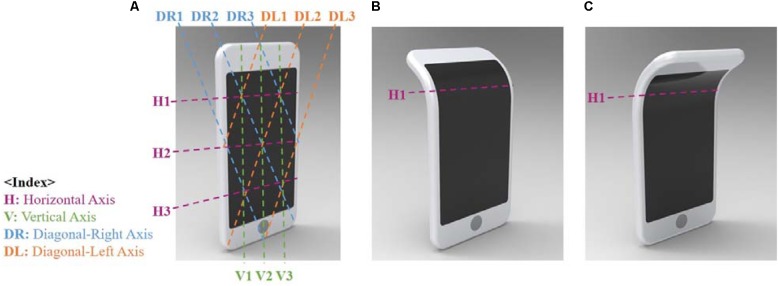
Shapes were made up of bends in three horizontal (H1, H2, H3), three vertical (V1, V2, V3), three diagonal right (DR1, DR2, DR3), and three diagonal left axes (DL1, DL2, DL3). **(A)** An example of flat shape (no bending), **(B)** an example of concave bending at a horizontal axis, **(C)** an example of convex bending at a horizontal axis.

In the current study, we used an online survey to investigate 281 users’ emotional evaluations regarding various shapes of an imaginary flexible device. The shape changes included folding, bending, rolling, pinching, zero-crossing, twisting, and crumpling. To generate these shapes, we computed all combinations of 60° bends in three horizontal, three vertical, three diagonal right, and three diagonal left axes (see **Figure 2**). For each axis, the device had one of three convexities: concave (bending forward), convex (bending backward), or flat (no bending). Strictly speaking, convex shape is often interpreted as either biconvex (both sides being curved outward) or plano-convex (single side being curved outward while the other side remains flat) while concave is interpreted as biconcave (both sides being curved inward) or plano-concave (single side being curved inward while the other side remains flat). However, as in Strohmeier et al. (2016), the convex shape in this study was a converging meniscus shape where the front side of the face curved outward while the opposite side of the face curved inward. Contrarily, the concave shape was a diverging meniscus shape where the front part of the face curved inward while the opposite face curved outward.

To eliminate redundancy in combinations (3^12^ = 531,441 shapes), the max number of bendings was three for 2049 shapes. Then, technically impossible shapes were discarded. These shapes included (1) shapes bent on both vertical and horizontal axes without diagonal bendings, (2) shapes bent on either vertical or horizontal axes as well as on diagonal axes, and (3) shapes bent on any diagonal-right axes as well as on the middle diagonal-left axis and vice versa. After eliminating technically impossible shapes, 153 shapes remained. Finally, vertically mirrored duplicates – 52 shapes – were again eliminated. As a result, 101 unique shapes were generated. All data and materials are publicly available on the project page in the Open Science Framework^1^.

### 3D Modeling

**FIGURE 3:**
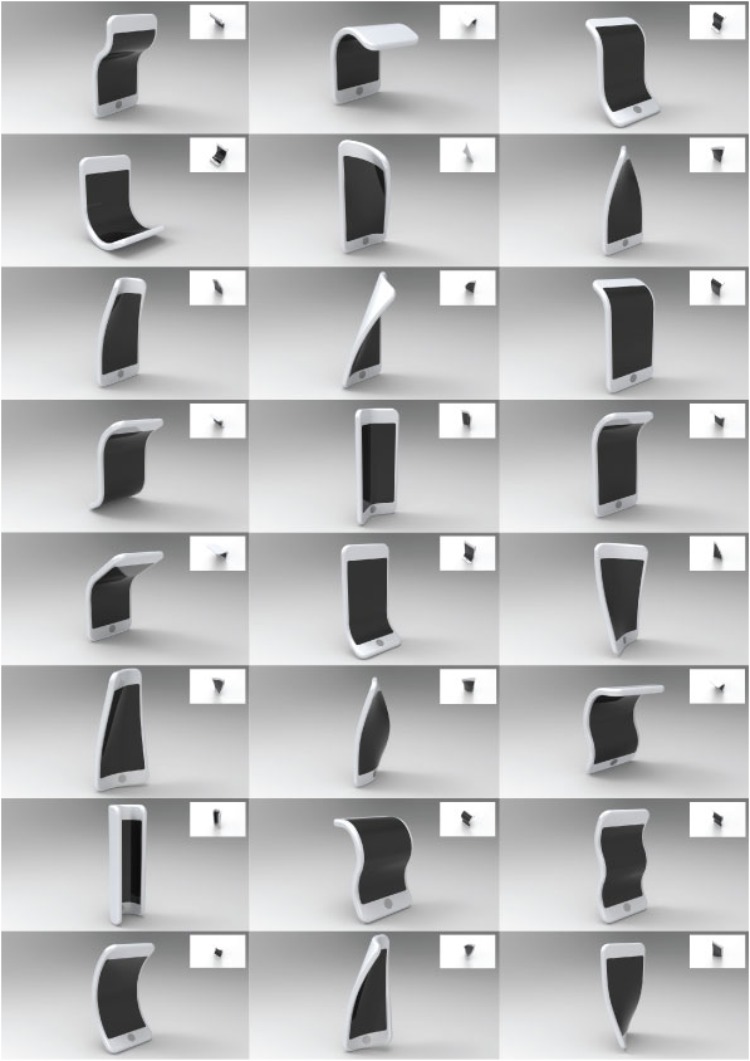
Examples of 3D modeled images. The main images of the shapes are positioned in the center, and the supplemental images of the shapes are placed at the top right corner.

These 101 possible 3D images of flexible displays were created using 3D Rhino. All the flexible displays featured an iPhone that was 67 mm × 138 mm × 7 mm. To remove the ambiguity that often occurs with a single viewpoint, each shape was rendered in two viewpoints – “distance = 266.12 mm, azimuth = -50°, Inclination = 15°” and “distance = 266.12 mm, azimuth = -132.53°, inclination = 53.22” – with the former as a main image positioned in the center and the latter as an additional image placed at the top right corner of the former (see **Figure 3**).

### Procedure and Measurements

A quantitative online survey was conducted for this study. After a short questionnaire for demographic information, all participants completed five blocks of emotional evaluation. In each block, 101 shapes were evaluated in terms of the targeted emotion: happiness, sadness, anger, disgust, and fear. For example, participants were asked to answer, “How much does the object look ANGRY?” for each of the 101 shapes in the first block, “How much does the object look HAPPY?” for the same shapes again in the second block, and so on. Participants responded by selecting a choice on a seven-point Likert-type scale (1 = not at all, 7 = very strongly). The block order was shuffled across participants, and shapes were presented in a random order in each block. The survey session took approximately 60–90 min. This study was carried out in accordance with the recommendations of the Institutional Review Board at Yonsei University. The protocol was approved by the Yonsei University Institutional Review Board. All participants, aged 18 and above, gave written informed consent in accordance with the Declaration of Helsinki. Participants received $10 in compensation for their participation and could quit anytime during the survey if they did not want to continue. No personal identifying information was collected.

## Results

### Participants

A total of 368 participants volunteered to participate in the study. To obtain data from the general population, we attempted to assign an equal quota across age groups and gender. None of the age–gender groups exceeded 10 ± 2.5% (see **Table 1**). Low reliability has often been pointed out as one of the major limitations of a web-based survey. To avoid this issue, we excluded outliers with three criteria. First, each emotion block included five checksum questions: five shapes were asked twice, randomly interleaved with the other 101 shapes for each emotion block. Participants were excluded if they gave different responses to the same question on two occasions (root mean squared error for 25 checksum questions was greater than 2.0). The first criterion detected 17 outliers. The second criterion was the max frequency in each block. Some participants could habitually give the same response for the most questions. Such outliers were detected by the max frequency; if a participant’s max frequency was greater than or equal to 101 (out of 106 questions including the checksum questions) in any emotional block (e.g., responding 3-3-3-3-3-… repeatedly 101 or more times), the participant was excluded. This criterion detected 25 outliers. With the second criterion, we could fail to find outliers who regularly changed answers (e.g., responding 3-4-3-4-3-4… repeatedly). To exclude these outliers, we also computed a standard deviation over 530 answers (106 answers × 5 emotional blocks) for each participant. If a participant’s SD was less than 1.0, we regarded the participant as an outlier. Following this rule, there were 58 outliers. In total, 87 outliers were detected and excluded in the following data analysis. Please note that some outliers belonged to two or more criteria. Responses for the checksum questions were also discarded in the analysis. Therefore, 101 responses for each block from the remaining 281 participants were analyzed in this study.

**Table 1:** Demographics of survey participants.

Age	Gender		Total (%)
	Male (%)	Female (%)	
10s	27 (9.09)	34 (11.45)	61 (20.54)
20s	30 (10.10)	27 (9.09)	57 (19.19)
30s	25 (8.42)	30 (10.10)	55 (18.52)
40s	26 (8.75)	31 (10.44)	57 (19.19)
50s and above	30 (10.10)	37 (12.46)	67 (22.56)
Total	138 (46.5)	159 (53.5)	297 (100)

### Correlation Analysis for Each Emotion

Although five elementary emotions on human facial expression have been identified (Ekman, 1999), little is known about the evaluation for the emotion of an object. To figure out the elementary emotions attributed to the flexible device, we tested correlations between the average scores (over 281 participants) for 101 images for two different emotions. As shown in **Table 2**, all emotional categories were significantly correlated except for between happy and anger. As expected, happiness showed significantly negative correlations with sadness (*r* = –0.256, *p* = 0.010), fear (*r* = –0.271, *p* = 0.006), and disgust (*r* = –0.274, *p* = 0.006) but a marginally significant negative correlation with anger (*r* = –0.184, *p* = 0.065). Among correlations between four negative emotions, sadness–fear (*r* = 0.954, *p* < 0.001) and anger–disgust (*r* = 0.904, *p* < 0.001) showed strong correlations.

**Table 2:** Correlation analysis of the emotions.

	Happiness	Sadness	Fear	Anger	Disgust
Happiness	1.000	-0.256^∗∗^	-0.271^∗∗^	-0.184	-0.274^∗^
Sadness		1.000	0.954^∗∗∗^	0.436^∗∗∗^	0.659^∗∗∗^
Fear			1.000	0.554^∗∗∗^	0.773^∗∗∗^
Anger				1.000	0.904^∗∗∗^
Disgust					1.000

*^∗^*p* < 0.05, ^∗∗^*p* < 0.01, ^∗∗∗^*p* < 0.001*.

To further investigate the relation between negative emotions, we tested the significance of the difference between correlation coefficients. As results, sadness had significantly greater correlation with fear than with anger (*z* = 9.85, *p* < 0.001) or disgust (*z* = 7.58, *p* < 0.001). Fear also had significantly greater correlation with sadness than with anger (*z* = 8.75, *p* < 0.001) or disgust (*z* = 5.93, *p* < 0.001). That is, correlation between sadness and fear was significantly stronger than any other correlations involving sadness and fear. Likewise, anger had significantly greater correlation with disgust than with sadness (*z* = 7.18, *p* < 0.001) or fear (*z* = 6.09, *p* < 0.001). Disgust also had significantly greater correlation with anger than with sadness (*z* = 4.92, *p* < 0.001) or fear (*z* = 3.26, *p* = 0.001). These results suggest that correlation between anger and disgust was significantly stronger than any other correlations involving anger and disgust.

To summarize, in contrast to the five emotions for facial expressions, participants perceived shapes of a flexible device as exhibiting three groups of emotions: (1) happiness, (2) sadness–fear, and (3) anger–disgust. In the following analysis, therefore, we will use three emotional categories. The sadness–fear score was calculated by averaging each participant’s sadness and fear ratings for each shape. Ratings for anger and disgust were also collapsed to produce the anger–disgust score.

### Pattern of Emotional Interaction

**FIGURE 4:**
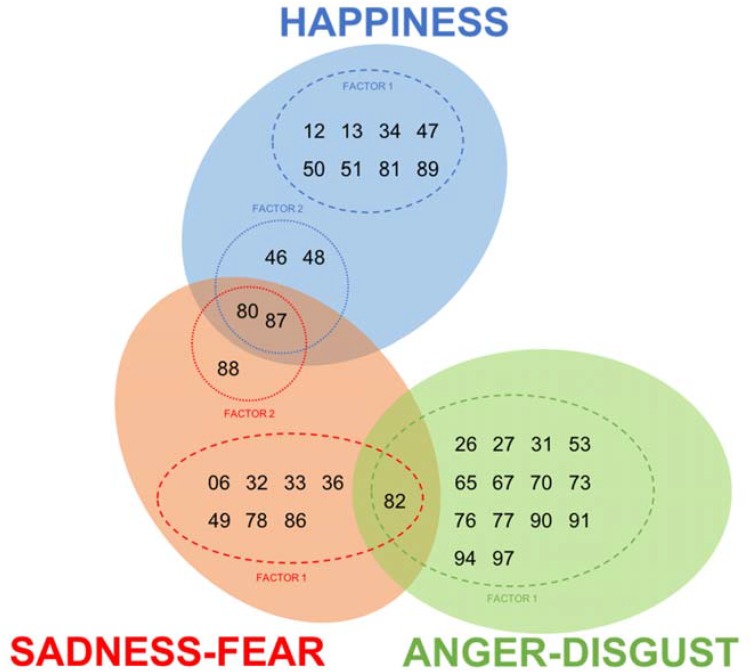
Highly emotional shapes for each emotional category. Numbers in the Venn diagram represent the image ID number. A total of 12, 11, and 15 shapes were selected for happy (blue circle), sad–fearful (red circle), and angry–disgusted (green circle), respectively. Only a few shapes evoked high emotional responses in two categories, and none did in all three categories. Dashed circles show factors in each emotional category. (See texts in the next section for more details).

To explore which shapes evoked strong emotional responses, we first selected shapes with an average rating higher than the mean + 1 SD of 101 rating scores (averaged across all 281 participants) for each emotional category. With criteria of 3.585 (=3.317 + 0.26), 3.992 (=3.537 + 0.455), and 4.000 (=3.650 + 0.350), a total of 12, 11, and 15 shapes were selected for happiness, sadness–fear, and anger–disgust, respectively. As clearly shown in **Figure 4**, only a few shapes evoked high emotional responses in two categories, and none did in all three categories.

#### Happiness

**FIGURE 5:**
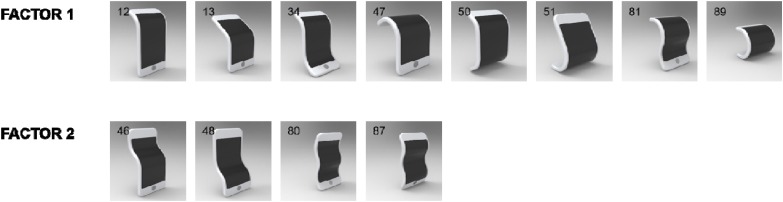
Shapes evoking happiness. All these shapes had bendings on horizontal axes (mainly convex) but not vertical or diagonal axis. Exploratory factor analysis suggested two factors for happiness. Numbers in the upper-left corner of images represent the shape ID numbers.

All high-happiness shapes had bendings on horizontal axes (mainly convex); none had a bending on vertical or diagonal axis. To further identify common factors of these shapes, we conducted an exploratory factor analysis with varimax rotation (*n* = 281). Results suggested there were two factors whose eigenvalues were greater than 1.0 (eigenvalue for factor 1 = 6.024; eigenvalue for factor 2 = 1.294), accounting for 60.984% of the total variance. Factor loadings of highly happy shapes and their physical properties are summarized in **Table 3**, and images for shapes are shown in **Figure 5**. The table and figure clearly show the unique property of the first factor: a convex bending on horizontal axes. All these shapes had one or two convex bending(s) at the middle or the top horizontal axis, reminding us a laughing figure. The second factor was made up of combinations of convex and concave bendings on horizontal axes in a sandwiched manner (e.g., concave-convex-concave) with either a simple (#46 and #48 with two bendings) or a complex shape (#80 or #87 with three bendings). These shapes reminded us a giggling figure.

**Table 3:** Factor loadings of 12 high-happiness shapes along with physical properties.

Shape ID	Factor 1	Factor 2	Bending											
			V1	V2	V3	H1	H2	H3	DR1	DR2	DR3	DL1	DL2	DL3
51	0.783		–	–	–	–	–1	–1	–	–	–	–	–	–
47	0.749		–	–	–	–1	–1	–	–	–	–	–	–	–
12	0.717		–	–	–	–1	–	–	–	–	–	–	–	–
50	0.715		–	–	–	–1	–	–1	–	–	–	–	–	–
89	0.705		–	–	–	–1	–1	–1	–	–	–	–	–	–
13	0.702		–	–	–	–	–1	–	–	–	–	–	–	–
81	0.602	0.546	–	–	–	–1	–1	1	–	–	–	–	–	–
34	0.524		–	–	–	–1	–	1	–	–	–	–	–	–
80		0.844	–	–	–	1	–1	1	–	–	–	–	–	–
87		0.836	–	–	–	–1	1	–1	–	–	–	–	–	–
46		0.655	–	–	–	1	–1	–	–	–	–	–	–	–
48		0.640	–	–	–	–	1	–1	–	–	–	–	–	–

*Factor loadings with absolute values less than 0.50 have been suppressed. Bending columns show convexity of bending on 12 possible lines (1 = concave; –1 = convex; dash = no bending)*.

#### Sadness–Fear

There were 11 shapes that participants rated highly sad and fearful. Except for shape #82, all highly sad–fearful shapes had bendings on horizontal axes (mainly concave); none had a bending on vertical or diagonal axis.

**FIGURE 6:**
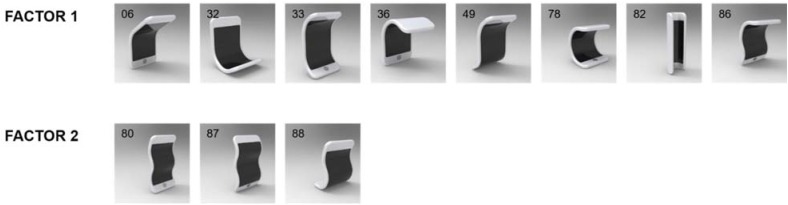
Shapes evoking sadness–fear. Except #82, all shapes had bendings on horizontal axes (mainly concave), but not a vertical or diagonal axis. Exploratory factor analysis suggested two factors for sadness–fear. Numbers in the upper-left corner of images represent the shape ID numbers.

We submitted all shapes to an exploratory factor analysis with varimax rotation (*n* = 281). Results suggested a two-factor solution (eigenvalue for factor 1 = 7.052, eigenvalue for factor 2 = 1.011), explaining 73.307% of the total variance. Factor loadings of highly sad and fearful shapes and their physical properties are summarized in **Table 4**, and images for shapes are shown in **Figure 6**.

**Table 4:** Factor loadings of 11 high-sadness–fear shapes along with physical properties.

Shape ID	Factor 1	Factor 2	Bending											
			V1	V2	V3	H1	H2	H3	DR1	DR2	DR3	DL1	DL2	DL3
36	0.863		–	–	–	1	1	–	–	–	–	–	–	–
32	0.849		–	–	–	–	1	1	–	–	–	–	–	–
78	0.804		–	–	–	1	1	1	–	–	–	–	–	–
6	0.779		–	–	–	–	1	–	–	–	–	–	–	–
33	0.696		–	–	–	1	–	1	–	–	–	–	–	–
49	0.694		–	–	–	1	–	–1	–	–	–	–	–	–
86	0.681	0.559	–	–	–	1	1	–1	–	–	–	–	–	–
82	0.669		1	1	1	–	–	–	–	–	–	–	–	–
87		0.885	–	–	–	–1	1	–1	–	–	–	–	–	–
80		0.866	–	–	–	1	–1	1	–	–	–	–	–	–
88		0.596	–	–	–	1	–1	–1	–	–	–	–	–	–

*Factor loadings with absolute values less than 0.50 have been suppressed. Bending column shows convexity of bendings on 12 possible lines (1 = concave; –1 = convex; dash = no bending)*.

The table and figure show the unique property of the first factor: a concave bending on horizontal axes. In contrast to happiness, sadness and fear was evoked by shapes that were bent in a concave manner at either the middle or the top horizontal axis or both. These shapes looked like a figure inclining its head. Shape #82 was an exception to this common property of the first factor. All of its vertical axes were bent concavely, resembling a figure inclining or shrinking inward.

Just like the second factor of highly happy shapes, the second factor of highly sad and fearful shapes is made up of combinations of concave and convex bendings on horizontal axes in complex shapes (three bendings). Indeed, shapes #80 and #87 evoked high happiness as well as high sad–fearful. These belonged to the second factors of both highly happy and highly sad and fearful shapes. It seemed that the common shapes and their properties (i.e., a combination of concave and convex bendings in a sandwiched manner with a complex shape) were perceived sometimes as giggling figures and sometimes as trembling figures.

#### Anger–Disgust

There were 15 shapes that participants rated highly angry and disgusting. In contrast to happy and sad–fearful emotional shapes, which had bendings on horizontal axes but no diagonal axis, all highly angry and disgusting shapes, except for #82, bendings on diagonal axes and no horizontal axis (neither concave nor convex). No emotional response was evoked by bending on vertical axis in any of the three emotional categories.

**FIGURE 7:**
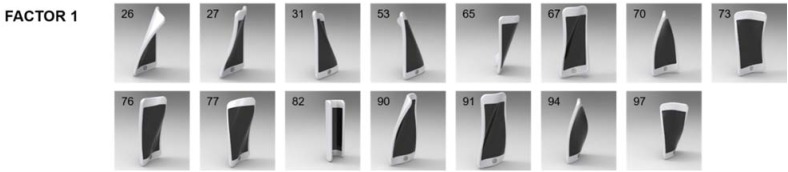
Shapes evoking anger–disgust. Except for shape #82, all shapes had bendings on diagonal axes but not vertical or horizontal axis. Exploratory factor analysis suggested there is a single factor for anger–disgust. Numbers in the upper-left corner of images represent the shape ID numbers.

We also conducted an exploratory factor analysis (*n* = 281) for high-anger–disgust shapes. Results showed that only the first factor had a dominant eigenvalue (eigenvalue for factor 1 = 9.283) compared to the remaining factors (eigenvalues for other factors <1.0). The first factor accounted for 61.885% of the total variance. Physical properties of high-anger–disgust shapes are summarized in **Table 5**, and images for shapes are shown in **Figure 7**.

**Table 5:** Physical properties of 15 high-anger–disgust objects.

Shape ID	Bending											
	V1	V2	V3	H1	H2	H3	DR1	DR2	DR3	DL1	DL2	DL3
26	–	–	–	–	–	–	–	–	–	1	1	–
27	–	–	–	–	–	–	–	–	–	–1	1	–
31	–	–	–	–	–	–	–	–1	1	–	–	–
53	–	–	–	–	–	–	–	–1	–1	–	–	–
65	–	–	–	–	–	–	–	–	–	–	–1	–1
67	–	–	–	–	–	–	–	–	–	–1	1	1
70	–	–	–	–	–	–	–	–	–1	1	–	1
73	–	–	–	–	–	–	–	–	1	–1	–	1
76	–	–	–	–	–	–	–	–	–	1	–1	1
77	–	–	–	–	–	–	–	–	–	–1	–1	1
82	1	1	1	–	–	–	–	–	–	–	–	–
90	–	–	–	–	–	–	–	–	–	1	1	–1
94	–	–	–	–	–	–	–	–	–1	1	–	–1
97	–	–	–	–	–	–	–	–	1	–1	–	–1
26	–	–	–	–	–	–	–	–	–	1	1	–

*Bending columns show convexity of bendings on 12 possible lines (1 = concave; –1 = convex; dash = no bending)*.

The table and figure show the unique property of high-anger–disgust shapes: bendings on diagonal axes. Except for shape #82, all these shapes had at least two bendings on diagonal axes. More specifically, they were always bent at the axes connected to the top edge of object (i.e., DL1, DL2, DR1, or DR2) at least once. Such a common property hinted to us that participants perceived these shapes as a figure twisting its shoulder.

Shape #82 was again an exception for this property. As shown in **Figure 4**, #82 belonged to high-anger–disgust shapes as well as high-sadness–fear shapes. It seemed that #82’s shape of shrinking its shoulder or body was often perceived as suggesting all negative emotional states.

## Discussion

### Categories of Emotion

In a series of studies, Ekman found that people – regardless of cultural background – categorized human facial expressions into five categories. In contrast, our study showed that people categorized objects’ shapes into three categories. Participants could not discriminate disgust from anger nor sadness from fear in our study. In some sense, it seems to be natural for sadness to be grouped with fear and anger with disgust. This is because, by nature, sadness and fear are directed inward (inside oneself) while anger and disgust are directed outward (toward an object outside of oneself).

High correlation between disgust and anger was commonly found in previous literature exploring recognition of emotions (Palermo and Coltheart, 2004; Tottenham et al., 2009; Recio et al., 2013). However, correlation between sadness and fear was not frequently reported. It is not clear why our participants did not show differences between two emotions. One possibility could relate to the precision of emotional expression. Facial expressions are made from combinations of 28 or more action units (facial muscles) with variable intensities, but our objects were generated with combinations of only eight units, each with a fixed intensity of bending. Another possible explanation is that participants perceived stimuli as postures rather than facial expressions. As Ekman pointed out, people are more sensitive to facial expressions than to the emotional value of postures. Future investigation will be required to clarify this issue.

### Relation Between Bending and Emotional Interaction

In the field of robotics, the cognitive underpinnings of emotional interaction between human and anthropomorphized robotics is considered crucial since robots have “synthetic psychology,” a state of not possessing internal emotion regardless of external emotional expression (Damiano and Dumouchel, 2018). That is, this new kind of synthetic interaction between human and anthropomorphized technology should be explored for the derivation of the emotionally interactive technologies considered necessary for social (Schmitz, 2011; Riether et al., 2012; Kwak, 2014) functionality, which enhance familiarity (Choi and Kim, 2009), likability (Castro-González et al., 2016), and encouragement (Breazeal, 2006).

This study found that the shape of the bended flexible display indicates a certain emotional expression, confirming the hypothesis that the anthropomorphic design of the flexible display would enable emotional interaction with the users. Since it is necessary to anthropomorphize the display using a simplistic pattern in the early stage of technical development, certain patterns that indicate emotion to users needed to be investigated. Here, based on the parameters that may underpin the emotional interaction of the flexible display, the axis of the bending and convexity of the curve were researched thoroughly. Through this empirical study, which examined how much the 3D-modeled image of a flexible display represents specific emotions to the participants, certain patterns have been found regarding the axis of bending and convexity. In terms of individual emotions, first, happiness was represented by the combination of a convexly curved display that has bendings on horizontal axes, significantly distinguishing itself from the other four emotions. Sadness and fear, which were highly correlated, had a concavely curved display that has bendings on horizontal axes. Last, anger and disgust, which also correlated with each other, had a curved display that has bendings on diagonal axes regardless of bending convexities.

Broadly, taking the conventional recognition concept that happiness conveys positive emotion while the other basic expressions, sadness, anger, fear, and disgust, are negative, it can be said that convex shape triggered positive emotion while concave shape conveyed negative emotion to the participants. This perception can be analyzed via Russell’s Circumplex model (Russell, 1980) since emotion space representation can be well presented through a plane defined by two dimensions. One of the indicators of this plane is the level of arousal while the other is the level of valence. Aligning with Jeong and Suk (2016), the result recognized the positivity and negativity of the valence on categorization of emotion. It was found that happiness, on the pleasant plane, has convex bending in a horizontal axes while other unpleasant emotions on the negative plane, anger, sadness, disgusted, and fear, have convex bending.

From this perspective, there is concern over misinterpretation of emotion expression since, regardless of subdivision of emotion group into categories, there are numerous ambiguous emotions. Indeed, according to Jack et al. (2014), it is necessary to find rules that trigger specific emotion since misinterpretation of emotional expression may impose negative UX to users, conveying social rejection to the user. Herein, it is necessary to find unique emotion since it reduces the chance of misinterpretation, demonstrating the necessity of future work to find other factors that influence clearer discrimination of emotional expression of flexible display in users.

The results in which the participants portrayed their emotional value on a flexible display align with the research conducted by Pedersen et al. (2014), Lee and Ju (2015), and Strohmeier et al. (2016), who investigated the emotional interaction of shape-changing displays. However, contrasting with Strohmeier et al. (2016), which found that bending on horizontal axis was the strongest predictor of the level of valence in the emotion, this research also highlights the significance of the curvature that bends on a diagonal axis, particularly when it conveys the emotions of anger and disgust.

### Parameters Underpinning Anthropomorphic Design of Flexible Displays

Our findings suggested some basic parameters required for anthropomorphic design of an emotional object. First, bending in horizontal and diagonal axes should be available. Bending along the horizontal axis should be applicable to express happiness, sadness, and fear while bending along a diagonal axis would express anger and disgust. Interestingly, bending along the vertical axis was not a critical factor for triggering emotional interaction in the users. Second, both concave and convex bending should be feasible. The results revealed that convex bending is required to express happiness, concave bending for sadness and fear, and both concave and convex for anger and disgust.

### Research Implications

In our study, we explored the object characteristics that reflect specific emotion. This study provides insight for understanding the emotional interaction between a human and an anthropomorphic object. It has been empirically shown that there exists emotional interaction between human and flexible displays, and three emotional categories for anthropomorphic flexible display have been suggested (Lee et al., 2015; Strohmeier et al., 2016). In this paper, we suggest a systematic method for studying emotional interaction between a human and an anthropomorphic object.

Flexible displays have enormous potential, and many believe that they will be commercially viable in the near future. However, studies of the anthropomorphic design of flexible displays and their user interaction remain scarce. Our study aimed to provide significant information to researchers and designers who intend to develop emotionally interactive devices or designs.

### Limitations of the Current Study

Although we successfully found factors influencing emotional interaction between humans and flexible objects, this study has a few limitations. First, the study was conducted with participants from a single cultural background. Ekman (1999) found that emotional perception was cross-cultural for facial expression. However, little is known of the effects of cultural difference on emotional perception for anthropomorphic objects. Therefore, to generalize our results, a cross-cultural study should be conducted with the same research framework as ours. Second, the study was carried out with only 101 static shapes, which were systemically made of combinations of 12 axes of bending. However, these shapes were not a comprehensive set of the postures possible for an object. For example, one might imagine thousands of other shapes by considering the angle of bending (e.g., 0, 30, 60, and 90°). It was technically difficult to collect data for all these shapes in a single study, but there could be various variables worthy of examination. For the same reason, we limited the scope of the current study to static objects. One could easily think that emotional interaction might be affected by a number of variables regarding movements such as speed, angle, amplitude, radius, and area in motion. The principles of animation could also be adopted, conducting more in-depth evaluation on shapes that evoke emotions by focusing on shapes that were built from the previous studies. These should be investigated in future studies.

## Author Contributions

JML, JB, and DYJ conceived, designed, and conducted the study. All authors wrote, reviewed, and edited the manuscript.

## Conflict of Interest Statement

The authors declare that the research was conducted in the absence of any commercial or financial relationships that could be construed as a potential conflict of interest.

## Footnotes

1 https://osf.io/bruvk

## References

[B1] Ahmaniemi T. Kildal J. Haveri M. (2014). “What is a device bend gesture really good?” in *Proceedings of the SIGCHI Conference on Human Factors in Computing Systems*, Toronto, 3503–3512. 10.1145/2556288.2557306

[B2] Atkinson A. P. Dittrich W. H. Gemmell A. J. Young A. W. (2004). Emotion perception from dynamic and static body expressions in point-light and full-light displays. *Perception* 33 717–746. 10.1068/p5096 15330366

[B3] Bae Y. Yoon I. Jeong S. Han I. Han G. (2018). U.S. Patent Application No. 15/435523. Washington, DC: U.S. Patent and Trademark Office. 10.1068/p5096 15330366

[B4] Bailenson J. N. Yee N. Brave S. Merget D. Koslow D. (2007). Virtual interpersonal touch: expressing and recognizing emotions through haptic devices. *Hum. Comput. Interact.* 22 325–353. 10.1080/07370020701493509

[B5] Baron-Cohen S. Spitz A. Cross P. (1993). Do children with autism recognize surprise? *Res. Note Cogn. Emot.* 7 507–516. 10.1080/02699939308409202

[B6] Bates J. (1994). The role of emotion in believable agents. *Commun. ACM* 37 122–125. 10.1145/176789.176803

[B7] Berger C. R. Bradac J. J. (1982). *Language and Social Knowledge: Uncertainty in Interpersonal Relations.* London: Edward Arnold.

[B8] Breazeal C. (2006). Human-robot partnership. *IEEE Intell. Syst.* 21 79–81.

[B9] Calvo M. G. Lundqvist D. (2008). Facial expressions of emotion (KDEF): identification under different display-duration conditions. *Behav. Res. Methods* 40 109–115. 10.3758/BRM.40.1.109 18411533

[B10] Castro-González Á. Admoni H. Scassellati B. (2016). Effects of form and motion on judgments of social robots× animacy, likability, trustworthiness and unpleasantness. *Int. J. Hum. Comput. Stud.* 90 27–38. 10.1016/j.ijhcs.2016.02.004

[B11] Choi J. Kim M. (2009). “The usage and evaluation of anthropomorphic form in robot design,” in *Proceedings of Undisciplined! Design Research Society Conference* Vol. 2008 Bath, 16–19.

[B12] Chouchourelou A. Matsuka T. Harber K. Shiffrar M. (2006). The visual analysis of emotional actions. *Soc. Neurosci.* 1 63–74. 10.1080/17470910600630599 18633776

[B13] Clarke T. J. Bradshaw M. F. Field D. T. Hampson S. E. Rose D. (2005). The perception of emotion from body movement in point-light displays of interpersonal dialogue. *Perception* 34 1171–1180. 10.1068/p5203 16309112

[B14] Damiano L. Dumouchel P. (2018). Anthropomorphism in human–robot co-evolution. *Front. Psychol.* 9:468. 10.3389/fpsyg.2018.00468 29632507 PMC5879791

[B15] Darwin C. (1872/1965). *The Expression of Emotions in Man and Animals.* London: John Marry.

[B16] Dawson J. Q. Schneider O. S. Ferstay J. Toker D. Link J. Haddad S. (2013). “It’s alive!: exploring the design space of a gesturing phone,” in *Proceedings of Graphics Interface*, Sascatchewan, 205–212. 10.20380/GI2013.27

[B17] Ekman P. (1965). Differential communication of affect by head and body cues. *J. Pers. Soc. Psychol.* 2 726–735. 10.1037/h0022736 5838771

[B18] Ekman P. (1999). “Basic emotions,” in *Handbook of Cognition and Emotion*, eds Dalgleish T. Power M. (New York,NY: John Wiley & Sons Ltd.), 45–60.

[B19] Epley N. Waytx A. Cacioppo J. T. (2007). On seeing human: a three-factor theory of anthropomorphism. *Psychol. Rev.* 114 864–886. 10.1037/0033-295X.114.4.864 17907867

[B20] Frijda N. (1988). The laws of emotion. *Am. Psychol.* 43 349–358. 10.1037/0003-066X.43.5.349 3389582

[B21] Gomes A. Nesbitt A. Vertegaal R. (2013). “MorePhone: a study of actuated shape deformations for flexible thin-film smartphone notifications,” in *Proceedings of the SIGCHI Conference on Human Factors in Computing Systems*, Paris, 583–592. 10.1145/2470654.2470737

[B22] Hemmert F. Lowe M. Wohlauf A. Joost G. (2013). “Animate mobiles: proximacally reactive posture actuation as a means of relational interaction with mobile phones,” in *Proceeding of Seventh International Conference on Tangible, Embedded and Embodied Interaction*, Barcelona, 267–270. 10.1145/2460625.2460669

[B23] Jack R. E. Garrod O. G. Schyns P. G. (2014). Dynamic facial expressions of emotion transmit an evolving hierarchy of signals over time. *Curr. Biol.* 24 187–192. 10.1016/j.cub.2013.11.064 24388852

[B24] James W. (1890). *The Principles of Psychology.* NewYork, NY: Henry Holt and Company.

[B25] Jellema T. Perrett D. I. (2003). Perceptual history influences neural responses to face and body postures. *J. Cogn. Neurosci.* 15 961–971. 10.1162/089892903770007353 14614807

[B26] Jeong K. A. Suk H. J. (2016). Affective effect of video playback and its assessment tool development. *Korean J. Sci. Emo. Sensibil.* 19 103–120.

[B27] Kahn P. Ishiguro H. Friedman B. Kanda T. (2006). “What is a human? toward psychological benchmarks in the field of human-robot interaction,” in *Proceeding of the 15th IEEE International Symposium on Robot and Human Interactive Communication*, New York, NY, 364–371.

[B28] Khalilbeigi M. Lissermann R. Kleine W. Steimle J. (2012). “FoldMe: interacting with double-sided foldable displays,” in *Proceedings of the Sixth International Conference on Tangible, Embedded and Embodied Interaction*, New York, NY, 33–40. 10.1145/2148131.2148142

[B29] Kwak S. S. (2014). The impact of the robot appearance types on social interaction with a robot and service evaluation of a robot. *Arch. Des. Res.* 27 81–93.

[B30] Kwon J. Y. Ju D. Y. (2018). Interior design of fully autonomous vehicle for emotional experience: focused on consumer’s consciousness toward in-vehicle activity. *Korean J. Sci. Emo. Sensibil.* 21 17–34. 10.14695/KJSOS.2018.21.1.17

[B31] Lee J. M. Ju D. Y. (2015). Personalization through personification: factors that influence personification of handheld devices. *Int. Conf. Hum. Comput. Interact.* 9170 440–447. 10.1007/978-3-319-20916-6-41

[B32] Lee J. M. Jeong S. Y. Ju D. Y. (2015). “Emotional interaction and notification of flexible handheld devices,” in *Proceedings of the 33rd Annual ACM Conference Extended Abstracts on Human Factors in Computing Systems*, Seoul, 2025–2030.

[B33] Lee J. M. Jeong S. Y. Ju D. Y. (2017). Variables that influence emotional interaction between human and personified flexible devices. *Int. J. Control Autom.* 10 15–26. 10.14257/ijca.2017.10.3.02

[B34] Lee S. Kim S. Jin B. Choi E. Kim B. Jia X. (2010). “How users manipulate deformable display as input device,” in *Proceedings of the SIGCHI Conference on Human Factors in Computing Systems*, Atlanta, 1647–1656.

[B35] Li J. Chignell M. (2011). Communication of emotion in social robots through simple head and arm movements. *Int. J. Soc. Robot.* 3 125–142. 10.1007/s12369-010-0071-x

[B36] Mathur V. Raj J. Chouhan K. Thanvi V. (2013). Nokia morph technology. *Int. J. Eng. Res. Tech.* 2 34–38.

[B37] Mone G. (2013). The future is flexible displays. *Commun. ACM* 56 16–17. 10.1145/2461256.2461263

[B38] Noda M. Kobayashi N. Katsuhara M. Yumoto A. Ushikura S. Yasuda R. (2011). An OTFT-driven rollable OLED display. *J. Soc. Inf. Disp.* 19 316–322. 10.1889/JSID19.4.316

[B39] Oatley K. Johnson-Laird P. (1987). Towards a cognitive theory of emotions. *Cogn. Emot.* 1 29–50. 10.1080/02699938708408362

[B40] Palermo R. Coltheart M. (2004). Photographs of facial expression: accuracy, response times, and ratings of intensity. *Behav. Res. Methods Instrum. Comput.* 36 634–638. 10.3758/BF03206544 15641409

[B41] Park Y. W. Park J. Nam T. J. (2015). “The trial of bendi in a coffeehouse: use of a shape-changing device for a tactile-visual phone conversation,” in *Proceedings of the 33rd Annual ACM Conference on Human Factors in Computing Systems*, Seoul, 2181–2190. 10.1145/2702123.2702326

[B42] Pedersen E. W. Subramanian S. Hornbæk K. (2014). “Is my phone alive?: a large-scale study of shape change in handheld devices using videos,” in *Proceedings of the 32nd Annual. ACM Conference on Human Factors in Computing Systems*, Boston, MA, 2579–2588.

[B43] Recio G. Schacht A. Sommer W. (2013). Classification of dynamic facial expressions of emotion presented briefly. *Cogn. Emot.* 27 1486–1494. 10.1080/02699931.2013.794128 23659578

[B44] Reeves B. Nass C. (1996). *The Media Equation: How People Treat Computers, Television and New Media Like Real People and Places.* New York, NY: Cambridge University Press.

[B45] Rendl C. Kim D. Fanello S. Parzer P. Rhemann C. Taylor J. (2014). “FlexSense: a transparent self-sensing deformable surface,” in *Proceedings of the 27th Annual ACM Symposium on User Interface Software and Technology*, Honolulu, 129–138. 10.1145/2642918.2647405

[B46] Ribeiro T. Paiva A. (2012). “The illusion of robotic life: principles and practices of animation for robots,” in *Proceedings of the seventh annual ACM/IEEE Int. Conference on Human-Robot Interaction*, Boston, MA, 383–390.

[B47] Riether N. Hegel F. Wrede B. Horstmann G. (2012). “Social facilitation with social robots?,” in *Proceedings of the Seventh Annual ACM/IEEE International Conference on Human-Robot Interaction*, Boston, MA, 41–48.

[B48] Rothkopf F. R. Janis A. J. Dabov T. (2014). U.S. Patent No. 8787016. Washington, DC: U.S. Patent and Trademark Office.

[B49] Russell J. A. (1980). A circumplex model of affect. *J. Pers. Soc. Psychol.* 39 1161–1178. 10.1037/h0077714

[B50] Saldien J. Vanderborght B. Goris K. Van Damme M. Lefeber D. (2014). A motion system for social and animated robots. *Int. J. Adv. Robot. Sys.* 11 72 10.5772/58402

[B51] Samsung (2013). *Samsung Highlights Innovations in Mobile Experiences Driven by Components, in CES Keynote.* Available at: https://www.samsung.com/semiconductor/insights/news-events/samsung-highlights-innovations-in-mobile-experiences-driven-by-components-in-ces-keynote/

[B52] Schmitz M. (2011). “Concepts for life-like interactive objects,” in *Proceedings of the Fifth International Conference on Tangible, Embedded, and Embodied Interaction*, New York, NY, 157–164. 10.1145/1935701.1935732

[B53] Schutt R. K. (2011). *Investigating the Soc. World: The Process and Practice of Res.* Newbury Park, CA: Pine Forge Press.

[B54] Shao Y. El-Kady M. F. Wang L. J. Zhang Q. Li Y. Wang H. (2015). Graphene-based materials for flexible supercapacitors. *Chem. Soc. Rev.* 44 3639–3665. 10.1039/c4cs00316k 25898904

[B55] Softbank Robotics (2017). *Who is Pepper?* Available at: https://www.ald.softbankrobotics.com/en/cool-robots/pepper

[B56] Strohmeier P. Carrascal J. P. Cheng B. Meban M. Vertegaal R. (2016). “An evaluation of shape changes for conveying emotions,” in *Proceedings of the 2016 CHI Conference on Human Factors in Computing Systems*, San Jose, 3781–3792. 10.1145/2858036.2858537

[B57] Takahashi S. (1995). Aesthetic properties of pictorial perception. *Psychol. Rev.* 102 671–683. 10.1037/0033-295X.102.4.671 7480468

[B58] Takayama L. Dooley D. Ju W. (2011). “Expressing thought: improving robot readability with animation principles,” in *Proceedings of Human-Robot Interaction (HRI), 2011 6th ACM/IEEE International Conference*, Lausanne, 69–76.

[B59] The RobotCub Consortium (2017). *iCub.Org.* Available at: http://www.icub.org/

[B60] Thomas F. Johnston O. Thomas F. (1995). *The Illusion of Life: Disney Animation.* New York, NY: Hyperion.

[B61] Tottenham N. Tanaka J. W. Leon A. C. McCarry T. Nurse M. Hare T. A. (2009). The NimStim set of facial expressions: judgments from untrained research participants. *Psychiatry Res.* 168 242–249. 10.1016/j.psychres.2008.05.006 19564050 PMC3474329

[B62] Triberti S. Chirico A. La Rocca G. Riva G. (2017). Developing emotional design: emotions as cognitive processes and their role in the design of interactive technologies. *Front. Psychol.* 8:1773. 10.3389/fpsyg.2017.01773 29062300 PMC5640767

[B63] Turkle S. (2010). “In good company? On the threshold of robotic companions,” in *Close Engagements with Artificial Companions: Key Social, Psychological, Ethical and Design Issues*, ed. Wilks Y. (Amsterdam: John Benjamins Publishing Company), 3–10.

[B64] van Breemen A. J. N. (2004). “Bringing robots to life: applying principles of animation to robots,” in *Proceedings of Shapping Human-Robot Interaction Workshop Held at CHI*, Vienna, 143–144.

[B65] Warren K. Lo J. Vadgama V. Girouard A. (2013). “Bending the rules: bend gesture classification for flexible displays,” in *Proceedings of the SIGCHI Conference on Human Factors in Computing Systems*, Paris, 607–610. 10.1145/2470654.2470740

[B66] Wiese E. Metta G. Wykowska A. (2017). Robots as intentional agents: using neuroscientific methods to make robots appear more social. *Front. Psychol.* 8:1663. 10.3389/fpsyg.2017.01663 29046651 PMC5632653

[B67] Yohanan S. MacLean K. E. (2012). The role of affective touch in human-robot interaction: human intent and expectations in touching the haptic creature. *Int. J. Soc. Robot.* 4 164–180. 10.1007/s12369-011-0126-7

[B68] Zlotowski J. Proudfoot D. Yogeeswaran K. Bartneck C. (2015). Anthropomorphism: opportunities and challenges in human-robot interaction. *Int. J. Soc. Robot.* 7 347–360. 10.1007/s12369-014-0267-6

